# Splanchnicectomy for Pancreatic Cancer Pain

**DOI:** 10.1155/2014/941726

**Published:** 2014-04-27

**Authors:** Toshiro Masuda, Masafumi Kuramoto, Shinya Shimada, Satoshi Ikeshima, Kenichiro Yamamoto, Kenichi Nakamura, Hideo Baba

**Affiliations:** ^1^Department of Surgery, Kumamoto General Hospital, Japan Community Health Care Organization, 10-10 Tohri-cho, Yatsushiro, Kumamoto 866-8660, Japan; ^2^Department of Gastroenterological Surgery, Graduate School of Medical Sciences, Kumamoto University, 1-1-1 Honjo, Chuo-ku, Kumamoto 860-8556, Japan

## Abstract

Persistent pain is a serious problem that often contributes to a poor quality of life in pancreatic cancer patients. Medical management by opioid analgesics is often accompanied by side effects and incomplete pain relief. A celiac plexus block is a simple treatment which relieves pain, but the procedure demands a certain degree of proficiency and the duration of the effects obtained can be rather limited. Transhiatal bilateral splanchnicectomy achieves a certain denervation of splanchnic nerves, but it requires a laparotomy. Unilateral thoracoscopic splanchnicectomy is a minimally invasive procedure to cause definite denervation. Bilateral thoracoscopic splanchnicectomy is recommended for unsatisfactory cases or recurrent pain occurring after the initial unilateral splanchnicectomy. It is important to select the most suitable treatment depending on patients' actual medical state and the predicted outcomes.

## 1. Introduction

Persistent pain is a serious problem that often contributes to anorexia and a poor quality of life for pancreatic cancer patients [1]. Moreover, it has been suggested that continuous pain might shorten the survival of such patients [2, 3]. Therefore, controlling the pain would seem to be one of the major objectives that clinicians should pursue in the treatment of pancreatic cancer patients. Recently, many kinds of opioids or nonsteroidal anti-inflammatory drugs have been developed for this purpose. However, medical management by opioid analgesics is often accompanied by various side effects and incomplete pain relief [4]. In particular, the administration of narcotics results in the sedative effects on normal activities of daily living.

The greater, lesser, and least splanchnic nerves carry sympathetic pain innervation to the upper abdominal viscera, including the pancreas, from the 5th to 8th, 9th to 10th, and 11th thoracic ganglia, respectively [5]. Visceral pain arises from the stimulation of a celiac ganglion, which then sends a signal to the splanchnic nerves [6]. To treat the intractable pain caused by pancreatic cancer, different chemical therapies or mechanical neurolysis of the splanchnic nerves has been developed.

Celiac plexus nerve block (CPB) was first described by Kappis in 1914 [7]. A double-blind randomized controlled trial showed that intraoperative CPB by injecting alcohol on each side of the aorta at the level of the celiac axis, versus the same amount of saline placebo, significantly reduced the pain score of surviving patients, for up to six months of follow-up observations [3]. CPB is now mainly applied in a percutaneous way, and several studies have been reported on the effectiveness of CPB as well as on some of its adverse events [3, 8–11]. CPB is now being widely employed as a simple procedure which brings satisfactory pain relief, although the duration of this response may be limited [7].

As a mechanical neurolysis of splanchnic nerves, the left unilateral splanchnicectomy by laparotomy in patients with chronic pancreatitis was introduced by Mallet-Guy in 1942 [12]. Splanchnicectomy by thoracotomy was described by Sadar and Cooperman [13] and Stone and Chauvin [14]. Then, in 1993, unilateral thoracoscopic splanchnicectomy (TS) for pancreatic cancer pain control was described by Worsey et al. [15]. Nowadays, TS for pain relief of chronic pancreatitis is widely performed, and some reports have studied its main beneficial effects for pain reduction [5, 16, 17].

In this review article, we focused on mechanical splanchnicectomy as a treatment of intractable pain control for pancreatic cancer patients.

## 2. Transhiatal Bilateral Splanchnicectomy

The procedure for transhiatal bilateral splanchnicectomy (TBS) was first described by Sastre et al. and by us [18, 19]. After laparotomy, a vertical incision through the retroperitoneum and the crus of the diaphragm was made on the aorta. The right greater splanchnic nerve is located just to the right of the azygos vein, and the left greater splanchnic nerveis located in front of the lower left hemiazygos vein. After cutting both sides of the nerves, the transected crus of the esophagus and the retroperitoneum, they are then closed [19].

Sastre et al. reported on 51 patients treated with transhiatal bilateral splanchnicectomy for intractable pain caused by unresectable pancreatic cancer [18]. TBS alone was performed for 22 patients, and TBS with biliary or gastrointestinal bypass was performed in 29 cases. Forty patients experienced good pain reduction immediately after the TBS and 32 showed good results for 3 months.

We have previously reported on the beneficial effects of TBS [19]. TBS was performed on 9 pancreatic cancer patients, 1 chronic pancreatitis with hepatoma, and 1 postcholedochojejunostomy. In the 11 patients, the mean pain reduction percentage was 85% (60–100%). Although TBS necessitates a laparotomy, it is a simple and safe technique, which is useful for an accurate assessment for the resectability of the malignancy, and also allows for the addition of other needed abdominal operations such as a biliary and/or intestinal bypass.

We reviewed the previously published articles on TBS and the data is shown in Tables 1 and 2. TBS may be optimal for pancreatic cancer patients who are determined to be unresectable after laparotomy or for those who need bypass surgery as an additional operation.

## 3. Unilateral Thoracoscopic Splanchnicectomy

Worsey et al. [15] and Takahashi et al. [22] reported on the treatment of intractable pain reduction by unilateral TS for unresectable pancreatic cancer. The original reports concerning TS are reviewed and presented in Tables 3 and 4.

Lonroth et al. reported that unilateral TS treatment showed significant effectiveness for reducing the pain of 4 patients with pancreatic cancer, 1 with duodenal cancer, 3 chronic pancreatitis, and 1 portal vein thrombosis [23]. They used the visual analogue pain score to evaluate the degree of the patients' pain in the study. The mean visual analogue pain scores of all the patients at each point, before the treatment, immediately after, and after 3 months, were 8.1, 1.3, and 2.9, respectively. Those data indicated that unilateral TS could induce an adequate and long-acting pain reduction and spare patients from having to tolerate the unbearable pain caused by pancreatic cancer.

Pietrabissa et al. reported on 24 patients treated with unilateral TS [27]. Four TS procedures ended in technical failures due to pleural adhesions. One patient required a contralateral TS for right-sided back pain after the treatment by the left-sided TS. Despite the apparent successful effects in pain reduction, the recurrence of the pain of low intensity within 24 hours after TS was observed in 8 of 20 patients. The authors also assessed the quality of life after TS treatment by using the Nottingham Health Profile questionnaire, and significant improvement in each area was observed for at least 1 month after the TS treatment.

Leksowski graded the degree of pain reduction after unilateral left TS in 26 pancreatic cancer patients based on detailed scoring factors including worst pain, least pain, general activity, mood, walking ability, relations with other people, sleep, and the enjoyment of life [29]. This study clearly revealed the long-acting effectiveness of unilateral TS on improving pain relief and the quality of life.

Recently, there have been several reports on TS [6, 30, 34–36] that propose its safety and effectiveness in improving the quality of life of pancreatic cancer patients. However, the necessity of contralateral TS and the time span of the pain reduction due to the treatment still remain controversial and undetermined.

## 4. Bilateral Thoracoscopic Splanchnicectomy

The usefulness of Bilateral TS for pain reduction in cases with pancreatic cancer was examined by Lin et al. [21] and Cuschieri et al. [20] in 1994. The former performed bilateral TS on 14 patients to reduce severe pain due to an upper abdominal cancer [21]. Sufficient pain reduction was observed in most of the patients except for two. But back pain was not completely relieved in one esophageal cancer patient or in one pancreatic cancer with vertebral bone invasion.

Cuschieri et al. introduced a bilateral TS performed through a posterior thoracoscopic approach [20]. They investigated 8 patients with intractable pain due to pancreatic cancer (*n* = 3) and chronic pancreatitis (*n* = 5). They demonstrated that the posterior route provided an excellent visual exposure of the mediastinum, chest wall, and sympathetic and splanchnic nerves without using single lung anesthesia.

Ihse et al. reported bilateral TS in a prone position [25]. They investigated the effects of bilateral TS on pain reduction and pancreatic function (standard secretin test, basal serum glucose, plasma insulin, and C-peptide) in 23 patients with pancreatic cancer and 21 with chronic pancreatitis, concluding that bilateral TS was beneficial for achieving good pain control and it does not entail any manifest deterioration of the pancreatic functions. Bilateral TS in a prone position is one of the favorable candidates as a reliable method to reduce the intractable pain due to upper abdominal cancer.

Kang et al. reported on 21 upper abdominal cancer patients treated with bilateral TS [32]. They also investigated the anatomy of splanchnic nerves and the sympathetic chain in 26 embalmed Korean cadaveric specimens. A frequent communication occurred between the greater and the lesser splanchnic nerves, which were both commonly found above the surface of the diaphragm. They emphasized that surgeons should learn more about the abundant distribution of the splanchnic nerve fibers to prevent the incomplete interruption of splanchnic nerves.

## 5. Comparison of Unilateral and Bilateral Thoracoscopic Splanchnicectomy

Saenz et al. reported on 13 patients treated with unilateral TS and 11 with bilateral TS [28]. Although the authors did not offer detailed data, they intimated that bilateral TS yields higher success rates than unilateral TS in pain control management.

Giraudo et al. reported on combining TS and laparoscopic gastrojejunostomy as a palliative treatment for unresectable advanced pancreatic cancer patients with uncontrollable pain and gastric outlet obstruction (unilateral TS: 4, bilateral TS: 4, and bilateral and laparoscopic gastrojejunostomy: 2) [26]. The mean operative time was 63 min for unilateral TS, 86 min for bilateral TS, and 190 min for the combination of bilateral TS and laparoscopic gastrojejunostomy. The authors emphasized the feasibility and safety of the endoscopic palliative treatment for various adverse symptoms due to advanced pancreatic cancer. The order of merit of unilateral and bilateral TS was not mentioned.

Le Pimpec Barthes et al. described the effectiveness of contralateral TS as an additional treatment in cases of insufficient pain reduction after unilateral TS [24]. They performed the pain-reducing treatment of unilateral TS, unilateral TS with associated vagotomy, and consecutive bilateral TS for 20 unresectable pancreatic cancer patients. The secondary TS of the contralateral side was applied for patients who did not have sufficient pain reduction after unilateral TS and the ensuing results were good. Therefore, they concluded that bilateral TS need not to be initially performed.

Katri et al. investigated the pain reductive effects of left- and right-sided TS performed for 12 pancreatic cancer patients [33]. They applied right-sided TS for the right-sided dominant pain and left-sided TS for the central, bilateral, and left-sided dominant pain. They reported that 2 patients required contralateral TS because of pain recurrence. One of the patients had successful pain relief lasting until death (9 months), and in the other patient the recurrence of the pain appeared after a period of 12 months.

Bilateral TS is not necessarily recommended as an initial palliative treatment for intractable cancer pain. The left-sided TS is mainly applied as a unilateral procedure, though it may be better to select either the left or the right unilateral TS depending on the actual location of the pain. A contralateral TS is recommended if the initial unilateral TS is not effective or the recurrence of the pain appears.

## 6. Comparison of Celiac Plexus Block and Thoracoscopic Splanchnicectomy

Some studies compared the effectiveness of CPB and TS. Stefaniak et al. investigated the intensity of the pain, quality of life, and opioid intake for 35 patients treated with CPB and 24 with unilateral TS [31]. They concluded that both procedures provided similar efficacy, but that CPB was preferable for its lower invasiveness and for having more positive effects on the quality of life.

On the other hand, Johnson et al. compared the efficacy of bilateral CPB, bilateral TS, and appropriate medical management alone among 65 patients with pancreatic or upper abdominal cancer [4]. In this randomized controlled study, they concluded that CPB or TS would not achieve sufficient pain reduction, when compared with appropriate medical management alone.

## 7. Conclusions

In general, the prognosis for advanced pancreatic cancer patients is extremely poor. Therefore, normally it is quite difficult to predict whether or which splanchnicectomy will lead to significant pain reduction and contribute to the quality of life of the patients. CPB is a simple procedure which brings pain relief, but it requires proficient skills and the duration of its effects may be limited. TBS can achieve a certain denervation of splanchnic nerve, although it necessitates a laparotomy. TBS can be also used as an additional operation when abdominal surgery is required for patients. Unilateral TS is a lesser invasive method than TBS to achieve a certain denervation level. Bilateral TS may be recommended for unsatisfactory cases or recurrent pain after the initial unilateral TS. There are lots of modalities to treat the intractable pain of pancreatic cancer patients. It is very important to select the most appropriate treatment depending on the individual patients' actual medical condition and predicted outcomes.

## Figures and Tables

**Table 1:** Reported transhiatal bilateral splanchnicectomy.

Author	Journal	Year	Number of total patients	Number of cancer patients	Total procedures of splanchnicectomy	Approach	Side	Position
Sastre et al. [18]	Surgery	1992	Pancreatic cancer (*n* = 51)	51	51	Bilateral (*n* = 51)	Bilateral (*n* = 51)	Supine position

Shimada et al. [19]	Surg Today	1999	Pancreatic cancer (*n* = 9), chronic pancreatitis (*n* = 1), postcholedochojejunostomy (*n* = 1)	9	11	Bilateral (*n* = 11)	Bilateral (*n* = 11)	Supine position

**Table 2:** Results of reported transhiatal bilateral splanchnicectomy.

Author	Operation time (min)	Complications	Assessment of pain	Pain scores before/after surgery	Patients free of opioids (%)
Sastre et al. [18]	NR	Pneumothorax (*n* = 1), chylothorax (*n* = 1), splenic injury (*n* = 1)	Three-step scale	32 in good (after 3 months)	NR

Shimada et al. [19]	NR	Transient hypotension (*n* = 8), pleural damage (*n* = 5)	0–4 pain score	3.5/1.4 (after 2 months)	NR

**Table 3:** Reported thoracoscopic splanchnicectomy.

Author	Journal	Year	Number of total patients	Number of cancer patients	Total procedures of splanchnicectomy	Approach	Side	Position
Worsey et al. [15]	Br J Surg	1993	Pancreatic cancer (*n* = 1)	1	1	Unilateral (*n* = 1)	Left (*n* = 1)	Lateral position

Cuschieri et al. [20]	J R Coll Surg Edinb	1994	Pancreatic cancer (*n* = 3), chronic pancreatitis (*n* = 5)	3	8	Bilateral (*n* = 8)	Bilateral (*n* = 8)	Prone position

Lin et al. [21]	Eur J Surg Suppl	1994	Pancreatic cancer (*n* = 7), hepatocellular carcinoma (*n* = 2), cholangiocarcinoma (*n* = 2), gastric cancer (*n* = 1), colon cancer with liver metastasis (*n* = 1), esophageal cancer (*n* = 1)	14	14	Unilateral (*n* = 1), bilateral (*n* = 13)	Right (*n* = 1), bilateral (*n* = 13)	Lateral position

Takahashi et al. [22]	Surg Endosc	1996	Pancreatic cancer (*n* = 3)	3	3	Unilateral (*n* = 3)	Left (*n* = 3)	Lateral position

Lonroth et al. [23]	Eur J Surg	1997	Pancreatic cancer (*n* = 4), duodenal cancer (*n* = 1), chronic pancreatitis (*n* = 3), portal vein thrombosis (*n* = 1)	5	5	Unilateral (*n* = 5)	Left (*n* = 5)	Lateral position

Le Pimpec Barthes et al. [24]	Ann Thorac Surg	1998	Pancreatic cancer (*n* = 20)	20	24	Unilateral (*n* = 16), bilateral (*n* = 4)	Left (*n* = 15), Right (*n* = 1), Bilateral (*n* = 4)	Lateral position

Ihse et al. [25]	Ann Surg	1999	Pancreatic cancer (*n* = 23), chronic pancreatitis (*n* = 21)	23	44	Bilateral (*n* = 44)	Bilateral (*n* = 44)	Prone position

Giraudo et al. [26]	Ann Oncol	1999	Pancreatic cancer (*n* = 14)	14	16	Unilateral (*n* = 4), bilateral (*n* = 6)	Left (*n* = 2), right (*n* = 2), bilateral (*n* = 6)	Lateral position

Pietrabissa et al. [27]	Arch Surg	2000	Pancreatic cancer (*n* = 24)	24	25 (technical failure in 4)	Unilateral (*n* = 21)	Left (*n* = 15), right (*n* = 6)	Lateral position

Saenz et al. [28]	Surg Endosc	2000	Pancreatic cancer (*n* = 24)	24	35	Unilateral (*n* = 13), bilateral (*n* = 11)	Left (*n* = 13), bilateral (*n* = 11)	Lateral position

Leksowski [29]	Surg Endosc	2001	Pancreatic cancer (*n* = 26)	26	26	Unilateral (*n* = 26)	Left (*n* = 26)	Lateral position

Krishna et al. [6]	Journal of Pain and Symptom Management	2001	Pancreatic cancer (*n* = 1)	1	1	Unilateral (*n* = 1)	NR	NR

Lang-Lazdunski et al. [30]	Ann Thorac Surg	2002	Adrenal metastasis (*n* = 1)	1	1	Unilateral (*n* = 1)	Left (*n* = 1)	Lateral position

Stefaniak et al. [31]	EJSO	2005	Pancreatic cancer (*n* = 59)	59	24	Unilateral (*n* = 24)	Left (*n* = 24)	Lateral position

Kang et al. [32]	Am J Surg	2007	Pancreatic cancer (*n* = 15), hepatic cancer (*n* = 2), gallbladder cancer (*n* = 2), bile duct cancer (*n* = 1), gastric cancer (*n* = 1)	21	21	Bilateral (*n* = 21)	Bilateral (*n* = 21)	Lateral position

Katri et al. [33]	J Laparoendosc Adv Surg Tech A	2008	Pancreatic cancer (*n* = 12)	12	15 (technical failure in 1)	Unilateral (*n* = 10), bilateral (*n* = 2, continuous)	Left (*n* = 10), Right (*n* = 4)	Lateral position

Johnson et al. [4]	Pancreatology	2009	Pancreatic cancer (*n* = 57), gallbladder cancer (*n* = 3), bile duct cancer (*n* = 1), duodenal cancer (*n* = 1), unknown cancer (*n* = 3)	65	15	Bilateral (*n* = 15)	Bilateral (*n* = 15)	Prone position

Prasad et al. [34]	J Minim Access Surg	2009	Pancreatic cancer (*n* = 1)	1	1	Bilateral (*n* = 1)	Bilateral (*n* = 1)	Prone position

Śmigielski et al. [35]	Videosurgery and Other Miniinvasive Techniques	2011	Pancreatic cancer (*n* = 89)	89	121	Unilateral (*n* = 121)	NR	Lateral position

Tavassoli et al. [36]	Journal of Cardio-Thoracic Medicine	2013	Pancreatic cancer (*n* = 20)	20	20	Unilateral (*n* = 20)	Left (*n* = 20)	Lateral position

**Table 4:** Results of reported thoracoscopic splanchnicectomy.

Author	Operation time (min)	Complications	Assessment of pain	Pain scores before/after surgery	Patients free of opioids (%)
Worsey et al. [15]	80	NR	NR	Pain free (after 1 month)	NR

Cuschieri et al. [20]	NR	None	NR	NR	NR

Lin et al. [21]	NR	Transient hypotension (*n* = 3)	NR	NR	85.7

Takahashi et al. [22]	87	Transient hypotension (*n* = 1)	NR	NR	100

Lonroth et al. [23]	<60	Pneumonia (*n* = 1), transient intercostal neuralgia (*n* = 2)	Visual analog scale	8.6/1.8 (immediately after)/3.7 (after 3 months)	33 (after 3 months)

Le Pimpec Barthes et al. [24]	NR	Intermittent diarrhea (*n* = 2)	NR	NR	80

Ihse et al. [25]	NR	Bleeding (*n* = 4)	Visual analog scale	8/3 (after 1 week)	>50

Giraudo et al. [26]	Unilateral, 63; bilateral, 86	Pneumothorax (*n* = 1), cholangitis (*n* = 1)	NR	NR	83.3 (after mean 4 months)

Pietrabissa et al. [27]	25 ± 9	Pneumothorax (*n* = 1), transient intercostal neuralgia (common)	Visual analog scale	7.4 ± 1.7/0.6 ± 1.0 (after 1 day)	95 (after 3 months)

Saenz et al. [28]	Unilateral, 58 ± 22; bilateral, 93.5 ± 15.6	Intercostal neuralgia (*n* = 4), persistent plural effusion (*n* = 1), pneumothorax (*n* = 1)	Visual analog scale	8.5/3 (after 30 day)	84 (after 4 days)

Leksowski [29]	NR	Transient intercostal neuralgia (*n* = 4)	Numeric rating scale	Worst pain; 8.54/ 1.77 (after 7 days)/ 1.59 (1 month)/ 1.85 (2 months)/ 2.00 (3 months)/ 1.93 (4 months)/ 2.25 (5 months)/ 2.80 (6 months)	100 (until death)

Krishna et al. [6]	NR	NR	Numeric rating scale	Worst pain; 9/3 (after 3 days)	0 (after 2 weeks)

Lang-Lazdunski et al. [30]	<60	None	Visual analog scale	NR/5 (after 6 weeks)	0 (after 6 weeks)

Stefaniak et al. [31]	NR	Transient intercostal neuralgia (*n* = 1)	Visual analog scale	Effect size; 11.27 (after 2 and 8 weeks)	46.1% down of opioid consumption (after 8 weeks)

Kang et al. [32]	95	None	Numeric rating scale	8.52 ± 1.08/1.71 ± 1.10 (after 7 days)	52.4 ( after 7 days)

Katri et al. [33]	31 ± 12	Intercostal neuralgia (*n* = 2), transient pleural effusion (*n* = 1)	Visual analog scale	8.08 ± 1/0.58 ± 0.79 (1 day)/1.08 ± 0.8 (after 1 month)	91.7

Johnson et al. [4]	NR	Wound infection (*n* = 1), intraoperative bleeding from diaphragm (*n* = 1)	Brief pain, inventory pain score	Worst pain; 6.11 ± 3.05/5.07 ± 2.76 (after 2 weeks)/5.00 ± 3.03 (2 months)	25 (after 2 months)

Prasad et al. [34]	NR	NR	Visual analog scale	8/2 (immediate postoperative period)	NR

Śmigielski et al. [35]	32 ± 18	Pneumothorax (*n* = 2)	Visual analog scale	5.66/2.33 (after 7 days)/1.78(30 days)	NR

Tavassoli et al. [36]	NR	Pneumothorax (*n* = 1)	Visual analog scale	8.2 ± 1.2/1.4 ± 1 (after 1 day)/1.7 ± 1.5 (1 week)/2.9 ± 1.2 (1 month)/4 ± 0.9 (3 months)	75
